# Supplementary material for: Hybrid Models and Biological Model Reduction with PyDSTool
Source: PLoS Comput Biol. 2012 Aug 9;8(8):e1002628. doi: 10.1371/journal.pcbi.1002628 (PMC3415397; doi:10.1371/journal.pcbi.1002628)
Supplement: Text S4 — Complete source code for the PyDSTool package (version 0.88.120504). Includes API documentation and help files linking to web pages. This file is identical to the current public release on Sourceforge.net. (ZIP) [file pcbi.1002628.s004.zip › PyDSTool/html/PyDSTool.Toolbox.optimizers.line_search.barzilai_borwein_non_monotone_search-pysrc.html]

xml version="1.0" encoding="ascii"?


PyDSTool.Toolbox.optimizers.line\_search.barzilai\_borwein\_non\_monotone\_search


| Home | Trees | Indices | Help | PyDSTool |
| --- | --- | --- | --- | --- |

Package PyDSTool ::
Package Toolbox ::
Package optimizers ::
Package line\_search ::
Module barzilai\_borwein\_non\_monotone\_search

[hide private]

[frames] | no frames]

# Source Code for Module PyDSTool.Toolbox.optimizers.line\_search.barzilai\_borwein\_non\_monotone\_search

```
 1  ##class Function(object): 
 2  ##  def __call__(self, x): 
 3  ##    return (x[0] - 2) ** 2 + (2 * x[1] + 4) ** 2 
 4  ## 
 5  ##  def gradient(self, x): 
 6  ##    return numpy.array((2 * (x[0] - 2), 4 * (2 * x[1] + 4))) 
 7   
 8  import sys 
 9  sys.path.append('/home/dmitrey/scikits/openopt/scikits/openopt/solvers/optimizers') 
10   
11       
12  from line_search import CubicInterpolationSearch 
13  from numpy import * 
14  from numpy.linalg import norm 
15   


16 -def BarzilaiBorwein_nonmonotone(function, x0, df = None, maxIter = 1000):


17      x0 = asfarray(x0) 
18      lineSearch = CubicInterpolationSearch(min_step_size = 0.0001) 
19       
20     
21      #TODO: get gradtol as self.gradtol 
22      gradtol = 1e-6 
23       
24   
25      g0 = function.gradient(x0) 
26      state0 = {'direction' : g0} 
27      x1 = lineSearch(origin = x0, state = state0, function = function) 
28       
29      alpha_k = abs(x1 - x0).sum() / abs(g0).sum() 
30   
31   
32   
33      #default settings 
34      eps = 1e-5 #must be 0 < eps << 1 
35      M = 8 
36      rho = 0.5 # must be from (0,1) 
37      sigma1, sigma2  = 0.015, 0.8 # must be 0 < sigma1 < sigma2 < 1 
38      sigma = 0.015 
39      #alpha_min, alpha_max = 1e-6, 1.5 # should be alpha_min <= sigma <= alpha_max 
40      alpha_min, alpha_max = 0.015, 1e6 
41   
42      xk = x0.copy() 
43      last_M_iter_objFun_values = [] 
44       
45      for k in xrange(maxIter): 
46          gk = function.gradient(xk) 
47          if norm(gk) <= gradtol: break 
48           
49          fk = function(xk) 
50          last_M_iter_objFun_values.append(fk) 
51          if len(last_M_iter_objFun_values) > M: last_M_iter_objFun_values.pop() 
52          maxObjFunValue = max(last_M_iter_objFun_values) 
53           
54          if not alpha_min <= alpha_k <= alpha_max: alpha_k = sigma 
55          Lambda = 1.0 / alpha_k 
56           
57           
58          while True: 
59              if function(xk - Lambda * gk) < maxObjFunValue - rho * Lambda * dot(gk.T, gk):  
60                  Lambda_k = Lambda 
61                  yk = -Lambda_k * gk 
62                  xk += yk 
63                  break 
64              sigma = (sigma1+sigma2)/2 # here alg says "choose sigma from [sigma1, sigma2]" 
65              Lambda *= sigma 
66           
67          alpha_k = -dot(gk.T, yk) / (Lambda_k * dot(gk.T, gk)) 
68           
69      #state['gradient'] = gk #TODO: uncomment me! 
70      return xk

71   
72  if __name__ == '__main__': 


73 -    class Function:


74 -        def __call__(self, x): return ((x-arange(x.size))**6).sum()


75 -        def gradient(self, x): return 6*(x-arange(x.size))**5

76       
77      x0 = sin(arange(300)) 
78      fun = Function() 
79      x_opt = BarzilaiBorwein_nonmonotone(fun, x0) 
80      print x_opt 
81      print fun(x_opt) 
82
```

  


| Home | Trees | Indices | Help | PyDSTool |
| --- | --- | --- | --- | --- |

|  |  |
| --- | --- |
| Generated by Epydoc 3.0.1 on Fri May 4 15:24:18 2012 | http://epydoc.sourceforge.net |
